# Sex differences in familial forms of frontotemporal dementia

**DOI:** 10.1002/alz.092552

**Published:** 2025-01-03

**Authors:** Molly B Memel, Adam M. Staffaroni, Ignacio Illán‐Gala, John Kornak, Rowan Saloner, Anna M. VandeBunte, Emily W. Paolillo, Claire J. Cadwallader, Coty Chen, Tania F Gendron, Hilary W. Heuer, Leah K. Forsberg, Brad F. Boeve, Adam L. Boxer, Howard J. Rosen, Kaitlin B. Casaletto, ALLFTD Consortium

**Affiliations:** ^1^ University of San Francisco, San Francisco, CA USA; ^2^ Memory and Aging Center, UCSF Weill Institute for Neurosciences, University of California, San Francisco, San Francisco, CA USA; ^3^ Hospital de la Santa Creu i Sant Pau ‐ Biomedical Research Institute Sant Pau ‐ Autonomous University of Barcelona, Barcelona, Catalonia Spain; ^4^ University of California, San Francisco, San Francisco, CA USA; ^5^ Mayo Clinic, Jacksonville, FL USA; ^6^ Department of Neurology, Mayo Clinic, Rochester, MN USA; ^7^ Memory and Aging Center, UCSF Weill Institute for Neurosciences, San Francisco, CA USA

## Abstract

**Background:**

Sex differences in neurodegenerative diseases can impact accurate diagnosis and management. Emerging data suggest there may be sex differences in frontotemporal dementia (FTD) prevalence and clinical manifestation. However, prior studies lacked longitudinal assessment and were limited in capturing sex differences in earliest stages of disease (e.g., preclinical). We evaluated how sex impacts longitudinal clinical outcomes in individuals carrying autosomal dominant variants for FTD.

**Method:**

337 participants carrying pathogenic variants in *MAPT*, *GRN*, or *C9orf72* completed longitudinal evaluations (2.4 average visits, range = 1‐6), including neuropsychological and functional assessments, and plasma analyzed for neurofilament light chain (NfL). FTLD Clinical Dementia Rating Scale (FTLD‐CDR) classified disease stage at study entry (FTLD‐CDR = 0: asymptomatic; FTLD‐CDR = 0.5: MCI; FTLD‐CDR> 0.5: Dementia). Linear mixed‐effects models evaluated the effect of sex on longitudinal clinical trajectories, including the effect of disease stage at study entry.

**Result:**

Male and female carriers were similarly distributed with respect to baseline demographics and most clinical outcomes. However, women exhibited higher baseline NfL than men. Not accounting for disease stage, men and women did not show statistical differences on cognitive, functional, or NfL trajectories. However, when accounting for disease stage, there was a significant interaction between baseline disease stage, sex, and time on cognitive, NfL, and functional trajectories. While sex differences were not statistically significant among asymptomatic carriers, women with MCI/dementia evidenced more rapid cognitive, NfL and functional decline over time compared to men. To evaluate the role of pathology burden, we tested the interaction between baseline NfL, sex, and time on clinical outcomes stratified by disease stage. In asymptomatic participants, women showed an attenuated adverse relationship between baseline NfL and clinical trajectories compared to men. No statistically significant sex differences were evident in the relationship between baseline NfL and clinical outcomes in participants with MCI/dementia.

**Conclusion:**

In familial FTD, women evidenced steeper clinical declines than men after symptom onset. Accounting for pathology, baseline NfL was a stronger predictor of clinical decline in asymptomatic men compared to women. NfL may be a less sensitive prognostic tool for clinical progression in women with FTD, particularly early in disease. This may reflect early “cognitive resilience” to neurodegeneration in women.

